# MicroRNA binding mediated Functional sequence variant in 3′-UTR of DNA repair Gene *XPC* in Age-related Cataract

**DOI:** 10.1038/s41598-018-33071-0

**Published:** 2018-10-12

**Authors:** Xi Zou, Lihua Kang, Mei Yang, Jian Wu, Huaijin Guan

**Affiliations:** 1 Department of Ophthalmology, Affiliated Hospital of Nantong University, Nantong, Jiangsu China; 2 The Third People’s Hospital of Changzhou, Changzhou, Jiangsu China

## Abstract

DNA oxidative damage repair is strongly involved in the pathogenesis of age-related cataract (ARC). The sequence variants of in coding region of DNA repair genes have been shown to be associated with ARC. It is known that single nucleotide polymorphisms (SNPs) in the 3′-terminal untranslated region (3′-UTR) can alter the gene expression by binding with microRNAs (miRNAs). We hypothesize that SNP(s) in miRNA binding site of certain DNA oxidative damage repair genes might associate with ARC risk. We examined 10 miRNA binding SNPs in 3′-UTR of 7 oxidative damage genes and revealed the *XPC*- rs2229090 C allele was associated with nuclear type of ARC (ARNC) risk in Chinese population. The individuals with the variant G allele (CG and GG) of *XPC*- rs2229090 had higher *XPC* mRNA expression compared to individuals carrying CC genotype. The *in vitro* assay showed that luciferase reporter gene expression can be down regulated by hsa-miR-589-5p in cells transfected with rs2229090 C allele compared to G allele. These results suggested that the C allele of *XPC*-2229090 increase the risk with ARNC. The mechanism underlying might be due to the stronger interation of the C allele with hsa-miR-589-5p, resulting in lower *XPC* expression and DNA repair capability than the individuals carring G allele in lens.

## Introduction

Age-related cataract (ARC) is a multifactorial disease that is the major cause of blindness worldwide^1^. Studies demonstrated that genes and environmental factors including aging, gender, ultraviolet rays, ionizing radiation and chemicals attribute to ARC^2,3^. One of the mechanisms for the above interaction is that the process triggers DNA oxidative damage and further leads to age-related diseases including ARC^4^.

DNA oxidative damage and inefficient of DNA repair capacity in lens epithelial cells (LECs) have been shown to be associated with ARC pathogenesis^4–7^. The repair of DNA damage is a pivotal mechanism to keep the homeostasis in mammalian cells. Nucleotide excision repair (NER), base excision repair (BER), double-strand break repair (DSBR) and mismatch repair (MMR)^8^ repair the various types DNA damage. Most oxidative DNA damage are rapidly repaired by NER and DSBR pathways^9,10^. NER mainly clears bulky adducts caused by chemical agents. DSBR may be rectified by either homologous or no homologous recombination pathways^10^. There is strong association between oxidative damage repair gene disrupted by gene variation in coding region and ARC^5,11,12^. However, a few studies focus on the 3′-untranslated region (3′-UTR) variation of gene.

Gene variation include copy number variations (CNVs) and single nucleotide polymorphisms (SNPs)^13^. In human genome, the most abundant form of DNA variation are SNPs^13,14^. SNP exist in any regions of DNA including intron, coding and untranslated region^15^.

MicroRNAs (miRNAs) are a group of noncoding RNAs, mature miRNAs contain approximately 22 nucleotides. They mainly interact with the 3′-UTR of mRNAs and restraint the gene transcript or lead to the mRNA degradation to regulate gene mRNA level^16–18^. SNPs located at miRNA binding sites (miRSNPs) can affect the base pairing between miRNA and target mRNA^19^, then further regulate miRNA-mediated genes mRNA level. It has been reported that SNPs in miRSNPs can adjust the expression of target genes of age-related disease, including cancer^20,21^, hypertension^22,23^, Parkinson’s disease^24^, Alzheimer disease^25^.

In our previous study, we have reported some miRSNPs in DNA repair genes such as *ZNF350* is related to ARC^26^. In this study, we select genes involved in the two pathways for ARC association, including *XPA* and *XPC* in NER pathway and *ATR, XRCC5, XRCC4, RAD55* and *RAD54* in DSBR pathway^10,19^. In current case-control study, we selected 10 SNPs located in the 3′-UTR of these genes to testify the relationship between ARC and these SNPs. Subsequently, *in vitro* assays were used to reveal the function of the SNPs.

## Materials and Methods

### Study Participants

The study was approved by the Ethics Committee of Affiliated Hospital of Nantong University and conducted in compliance with the Declaration of Helsinki. All participants were told the purpose and obtained the Informed consent.

This nested case-control study included cases and controls from a population based epidemiologic cohort of the Jiangsu Eye Study located in Funing and Qidong counties. All participants were followed up with a series of ophthalmic evaluation including vision acuity, lens examination by a slit lamp biomicroscope after mydriasis, and ophthalmoscopic examination.

According to the opacity region of lens, the type of ARC was classified into four subtypes: cortical cataract (C), nuclear cataract (N), posterior sub capsular cataract (PSC) and mixed cataract (M)^27^. The Lens Opacities Classification System III (LOCSIII) was used to diagnose and grade lens opacities^28^. The age- and sex-matched controls who have transparent lens were also included from same communities. The criteria of our epidemiological investigation for the ARC group was LOCSIII > C2; >N2; >P2, while the control group was LOCSIII ≤ C1; ≤N1; ≤P1. The covered area of the study has a relatively stable and ethnically homogenous population. The details in the inclusion/exclusion of the case-control design were described in our previous study^11^. Consequently, 993 ARC patients (C = 453, N = 276, PSC = 52, M = 212) and 993 controls were included (Table 1).

**Table 1:** Demographic Information of Study Participants.

Variable	n	Age		*P*	Sex		χ^2^	*P*
		Mean ± SD	Range		Male (%)	Female (%)		
Controls	993	69.80 ± 4.47	50–80		447 (45.0)	546 (55.0)		
ARCs	993	69.72 ± 6.04	50–80	0.73	449 (45.2)	544 (54.8)	0.008	0.48
C	453	70.15 ± 5.64	52–80	0.45	201 (44.4)	252 (55.6)	0.052	0.52
N	276	68.98 ± 5.86	50–79	0.12	128 (46.4)	148 (53.6)	0.162	0.37
P	52	68.15 ± 4.16	50–78	0.09	24 (46.2)	28 (51.58)	0.026	0.49
M	212	70.14 ± 6.14	51–80	0.51	96 (45.1)	116 (54.7)	0.005	0.50

C: cortical; N: nuclear; P: posterior sub capsular cataract; M: mixed type.

To collect ocular tissue and matched veinal blood, additional 20 ARNC patients (LOCSIII > N2) and 20 age-, sex- and ethnically-matched controls from inpatients/outpatients of our hospital were recruited (Table S1). The average age is 65.8 ± 6.7 years in ARNC patients and 65.3 ± 6.4 years in controls. The ratio of sex is 0.55 in ARNC patients and 0.5 in controls. There is no difference between age and sex. The lens capsule samples were collected for measuring mRNA level and the rate of oxidative damage of LECs. The veinal blood was drawn for DNA genotyping and oxidative damage assessment of lymphocytes. All those ARNC patients’ capsule samples was harvested by phacoemulsification. The controls’ LECs from transparent lens were obtained from patients who had lens extraction during vitrectomy. We excluded the patients (both cases and controls) who had lens trauma, diabetes, uveitis glaucoma and high myopia (>6D) according our previous study^4^.

### DNA, RNA and cDNA preparation

Genomic DNA extraction from Veinal blood was used by Qiagen Blood DNA Mini Kit (Qiagen, Valencia, CA) according to the manufacturer’s instructions.

Total RNA was isolated by Trizol reagent from lens capsule samples (Invitrogen, Carlsbad, CA). Then cDNAs were performed by PrimeScript RT reagent Kit (TaKaRa, Dalian, China).

### Selection of SNPs and genotyping

Haplotype-tagging SNPs located in the 3′-UTR regions of some DNA repair genes were selected by searching Han Chinese data in NCBI dbSNP (https://www.ncbi.nlm.nih.gov/snp). The SNPs with a MAF ≥ 10% were included while excluding those having strong linkage disequilibrium (LD) between adjacent variants with r^2^ threshold ≤0.80 (Table 2).

**Table 2:** The Included SNPs of the 3′-UTR of the Selected Genes.

Gene name	Function in DNA repair	SNPs	Nucleotide change	MAF	miRNA binding
*XPA*	NER	rs3176752	C > A	0.10	hsa-miR-4753–3p
*XPC*	NER	rs2229090	C > G	0.29	hsa-miR-589-5p
*ATR*	DSBR	rs2241201	G > C	0.15	hsa-miR-4731-3p hsa-miR-4801
		rs877710	C > G	0.25	hsa-miR-564
		rs11067231	C > A	0.25	hsa-miR-1972
		rs11067233	C > G	0.10	hsa-miR-603
*RAD54*	DSBR	rs7310449	A > G	0.4	hsa-miR-299-3p
*RAD55*	DSBR	rs7301931	C > T	0.4	hsa-miR-4322
*XRCC4*	DSBR	rs2035990	C > T	0.5	hsa-miR-567
*XRCC5*	DSBR	rs2440	C > T	0.2	hsa-miR-548ao-3p

MAF: minor allele frequency in Chinese population.

SNP Genotyping was performed with the TaqMan genotyping assay (Thermos fisher, Foster City, CA, USA) according to the manufacturer’s instructions, as described in our previous publications^29,30^.

### *In silico* analysis

The PolymiRTS database 3.0 (http://compbio.uthsc.edu/miRSNP) and miRNA Target Detection (http://www.microrna.org/microrna/getGeneForm.do) were used to predict the candidate miRNAs which bind the selected 3′-UTR sequences. LD analysis was analyzed based on the 1000 Genomes data for the CEU population using the SNP Annotation and Proxy (SNAP) tool (http://www.broadinstitute.org/mpg/snap)^31^. An online software, the RNAhybrid program (http://bibiserv.techfak.uni-bielefeld.de/rnahybrid), was employed to calculate the minimum free energy (MFE) of hybridization between miRNAs and their potential target sequences with MFE < −20 kcal/mol as the threshold were selected according previous study^29,31^.

### Comet Assay

Comet assay, the single cell gel electrophoresis assay, is a sensitive technique to detect the DNA breaks. We measured DNA damage of LECs from the capsule samples and lymphocytes from peripheral venous blood using comet Assay kit (Trevigen, Gaithersburg, Maryland, USA) according to the manufacturer’s protocol. LECs and lymphocytes were isolated and then suspended at 1 × 10^4^ cells/ml in PBS. The data analysis was performed with measuring the percentage of DNA in the tail of comets (%Tail DNA) and the olive tail moment (OTM) according to the method described by our previous study^32,33^.

### Quantification of *XPC* mRNA expression

TaqMan gene expression assay probes (Thermos fisher) were used for *XPC* mRNA quantification (assay ID: Hs01104205_g1). Human GAPDH (Hs02786624_g1) was used as housekeeping gene control. Real-time PCR analysis was performed by ABI StepOne plus real-time PCR system (Applied Biosystems, Foster City, CA, USA). The fold change of genes mRNA level was calculated using 2^(−ΔΔCt)^ algorithm.

### Plasmids construct

The 3′-UTR of the *XPC* 2229090-C or *XPC* 2229090-G was cloned to pmiR-RB-REPORT™ vector (Ribobio, Guangzhou, China). The designed and synthesized primer of reporter construct (*XPC*-rs2229090) were as follows:

*XPC* -WT-f TCGAGCATGCCCAGCCCCTGGTGGTGGGGGCTTCTCTGCTGAGAAGGCAAACTGAGGC;

*XPC* -WT-r GGCCGCCTCAGTTTGCCTTCTCAGCAGAGAAGCCCCCACCACCAGGGGCTGGGCATGC;

*XPC* -MUT-f TCGAGCATGCCCAGCCCCTGGTGGTGGGGGGTTCTCTGCTGAGAAGGCAAACTGAGGC;

*XPC* -MUT-r GGCCGCCTCAGTTTGCCTTCTCAGCAGAGAACCCCCCACCACCAGGGGCTGGGCATGC.

The synthetic sequences of reporter construct (*XPC*-rs2229090) were as follows:

*XPC*-WT:

GGCGATCGCTCGAGCATGCCCAGCCCCTGGTGGTGGGGGCTTCTCTGCTGAGAAGGCAAACTG;

*XPC*-MUT:

GAGCATGCCCAGCCCCTGGTGGTGGGGGGTTCTCTGCTGAGAAGGCAAACTCGAGGCGGCCGCTGGCCGCAAT.

DNA sequencing was used to confirm the recombinant constructs.

### Cells culture and transfection

SRA01/04 cell line originated from human lens epithelium was bought from Chinese Academy of Sciences (Shanghai, China). The cells were cultured in Dulbecco’s modified eagle medium (DMEM) (Invitrogen) supplemented with 10% fetal bovine serum (FBS) (Lonza, Basel, Switzerland), 1% Penicillin-Streptomycin Solution (100 U/ml of penicillin and 0.1 mg/ml of Streptomycin) according our previous study^18^, in a humidified atmosphere with 5% CO_2_ at 37 °C. The cells were plated into 96-well plates at a density of 2 × 10^5^ cells/well. Transfection was conducted when cells reached 60–70% confluence.

The cells only transfected with miRNA mimics or inhibitors (Ribobio) were for qRT-PCR assays and co-transfected with miRNA mimics or inhibitors reporter and plasmids were for Luciferase reporter assay. We used riboFECT^TM^ Transfection kit (Ribobio) to transfect 100 ng/well reporter plasmids (*XPC* 2229090-C, *XPC* 2229090-G or Blank controls), 50 nmol/L hsa-miR-589-5p mimics, 100 nmol/L hsa-miR-589-5p inhibitors, or 50 nmol/L miRNA mimics controls, 100 nmol/L inhibitor controls into the cells, respectively.

### Luciferase reporter assay

The cells were prepared 48 h after transfection, and 100 μl of supernatants was removed from each well for luminescence assay. A luciferase assay kit (Promega, Madison, Wisconsin) was used to measure luciferase activity. Experiments were repeated at least three times.

### Statistical analysis

Statistical analyses were used by Stata software (Stata Corp, College Station, TX). The χ^2^ test was performed to test the association between the alleles frequencies of ARC groups and controls, and to calculate odds ratios (OR) and 95% confidence interval (CI). Hardy- Weinberg Equilibrium (HWE) of genotype distributions were also tested by the χ^2^ test. Bonferroni correction was conducted when positive association exist in the initial allele analysis. Various genetic model analyses were performed to characterize the association as dominant (mutant type homozygote versus wild type homozygotes and heterozygote), recessive model (heterozygotes and mutant type homozygotes versus wild type homozygotes). We only present the most significant model in the results. *P* < 0.05 was considered as statistically significant. The values of the Comet assay were expressed as mean ± SD. The ANOVA was used to compare the differences of the Comet assay parameters between the genotypes. *P* < 0.05 was considered as statistically significant. The qRT-PCR and Luciferase assay in this study were repeated at least 3 times independently. Data were presented as means ± SD. The *t* test was used to compare the average values of two groups.

## Results

### Characteristics of the participants for the association study

The participants of the study were recruited from the epidemiologic. The Table 1 showed the general demographic characteristic of the study participants. There was no statistically significant difference about age and gender between ARCs and controls (*P* > 0.05).

### Bioinformatics selection of candidate SNPs

Ten SNPs in 3′-UTR region of seven genes were selected for genotyping. Their basic information and predicted miRNAs were listed in Table 2.

### Association between SNPs and risk of ARC

Among the ten SNPs, the allele frequency of *XPC*-2229090 of ARC cases was significantly different from those of controls before and after multiple comparison correction (Bonferroni correction) (*P* < 0.0001, *Pa* < 0.001) (Table 3). We then further performed stratification analysis to explore the SNP involvement in subtypes of ARC. The results showed that frequency of the minor alleles of *XPC*-2229090 were significantly lower in the C, N and M type of ARCs than in the controls (*P* = 0.0372; *P* = 0.0001; *P* = 0.008) (Table 4). However, the significances of the SNPs was only present between ARNC and controls after Bonferroni correction.

**Table 3:** Summary of Associations between the SNPs and ARC.

Gene	SNPs Major/Minor	Controls Major/Minor	ARCs Major/Minor	χ^2^	*P/Pa*	OR (95%CI)
*XPA*	rs3176752 C/A	1740/246 (12.4%)	1751/235 (11.8%)	0.286	0.592	0.95 (0.78-1.15)
***XPC***	**rs2229090** **C/G**	1171/815 (41.3%)	1305/681 (34.3%)	19.255	**<0.001/<0.01**	0.75 (0.66–0.85)
*ATR*	rs2241201 G/C	1560/426 (21.5%)	1553/433 (21.8%)	0.073	0.787	1.02 (0.88–1.19)
	rs877710 C/G	1343/643 (32.4%)	1306/680 (34.2%)	1.552	0.213	1.09 (0.95–1.24)
	rs11067231 C/A	1350/636 (47.1%)	1343/643 (32.4%)	0.057	0.812	1.01 (0.89–1.16)
	rs11067233 C/G	1736/250 (12.6%)	1726/260 (13.1%)	0.225	0.635	1.05 (0.87–1.26)
*RAD54*	rs7310449 A/G	1191/795 (40.0%)	1172/814 (41.0%)	0.377	0.280	1.04 (0.92–1.18)
*RAD55*	rs7301931 C/T	1112/874 (44.0%)	1096/890 (44.8%)	0.261	0.316	1.03 (0.91–1.17)
*XRCC4*	rs2035990 C/T	1003/983 (49.5%)	1015/971 (48.9%)	0.145	0.364	0.98 (0.86–1.11)
*XRCC5*	rs2440 C/T	1589/397 (20.0%)	1567/419 (21.1%)	0.746	0.205	1.07 (0.92–1.25)

*Pa: P* value after Bonferroni correction.

**Table 4:** Association between rs2229090 and the C, N, M Type of ARC.

Gene/SNP	Allele	Control, n (%)	C Type of ARC, n (%)	N Type of ARC, n (%)	M Type of ARC, n (%)
*XPC*/rs2229090	C	1171 (58.96)	570 (62.91)	389 (70.47)	277 (65.33)
	G	815 (41.04)	336 (37.09)	163 (29.53)	147 (34.67)
*P*/*Pa*			0.044/0.44	**<0.0001/<0.001**	0.008/0.08
OR (95% CI)			0.85 (0.72–0.99)	0.60 (0.49–0.74)	0.76 (0.61–0.95)

The genetic model analysis found that rs2229090 were associated with the relevant types of ARC in the dominant model and the recessive model. The associations still exist after Bonferroni correction (*P* < 0.05) (Table 5).

**Table 5:** Association Between rs2229090 and the N Type of ARC.

Gene/SNP	Allele	Control, n (%)	N, n (%)	*P*/*Pa*	OR (95% CI)
*XPC*/rs2229090	C	1171 (58.96)	376 (68.12)	<0.0001/<0.001	0.67
	G	815 (41.04)	176 (31.88)		(0.55–0.82)
dominant model	CC	356 (35.85)	128 (46.38)	0.0014/0.014	0.65
	CG + GG	637 (64.15)	148 (53.62)		(0.49–0.85)
recessive model	CC + CG	815 (82.72)	248 (89.86)	0.0019/0.019	0.52
	GG	178 (17.93)	28 (10.14)		(0.34–0.79)

### The effects of rs2229090 on the mRNA levels of *XPC* in biopsy samples

**Figure 1:**
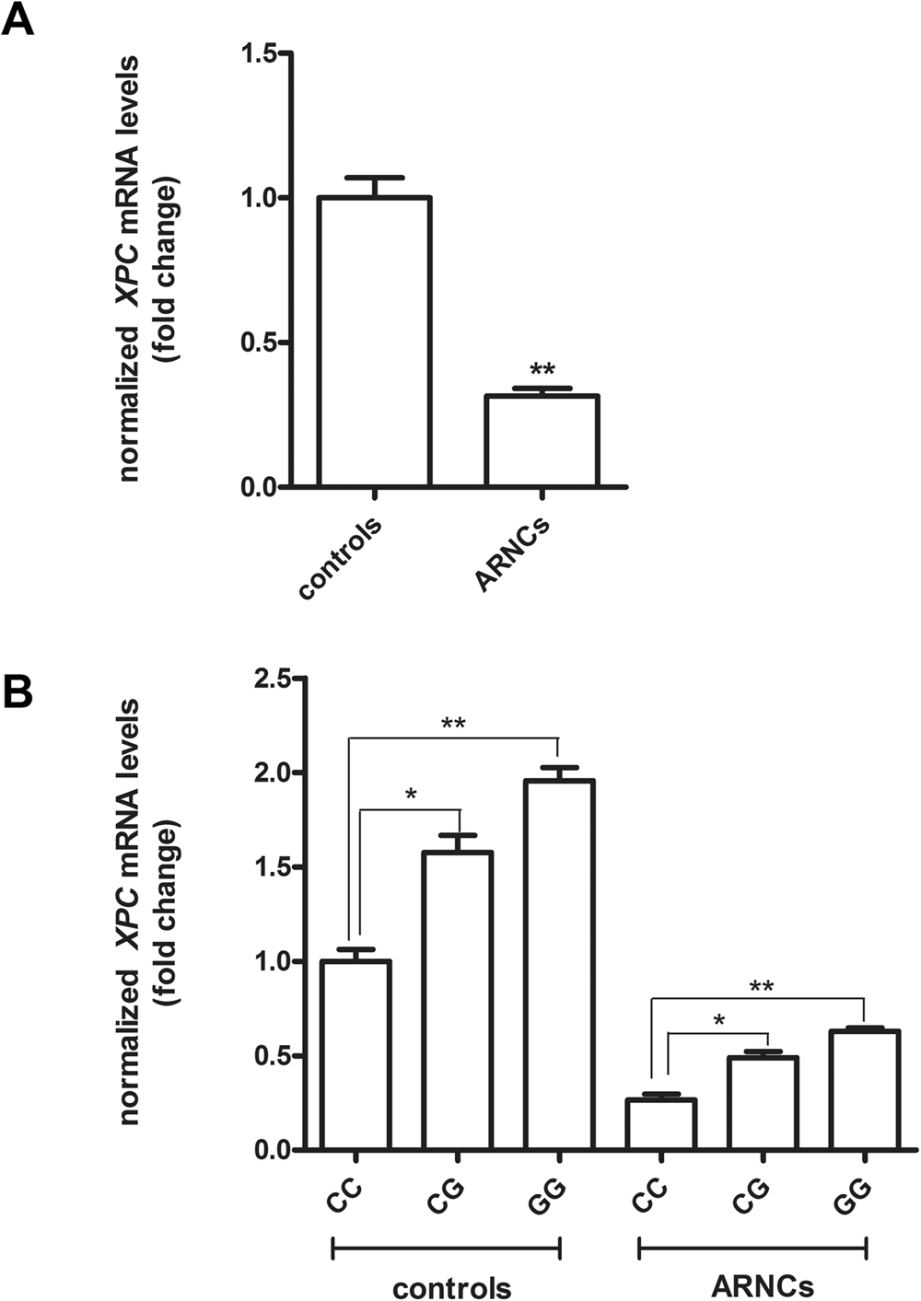
Levels of *XPC* mRNA expression in anterior capsules. (**A**) *XPC* mRNA levels were lower in ARNCs than the controls. (**B**) *XPC* mRNA levels were higher in CG or GG group than the CC group. *P < 0.05, **P < 0.01.

As shown in Fig. 1A, the mRNA expression of *XPC* was lower in LECs of ARNC group than that of the controls. Moreover, individuals carrying the minor G allele in all subjects compared with the CC genotype (CG versus CC, *P* < 0.05; GG versus CC, *P* < 0.01) (Fig. 1B).

### The correlation of rs2229090 with DNA breaks and ARC risk evaluated by Comet assay

**Figure 2:**
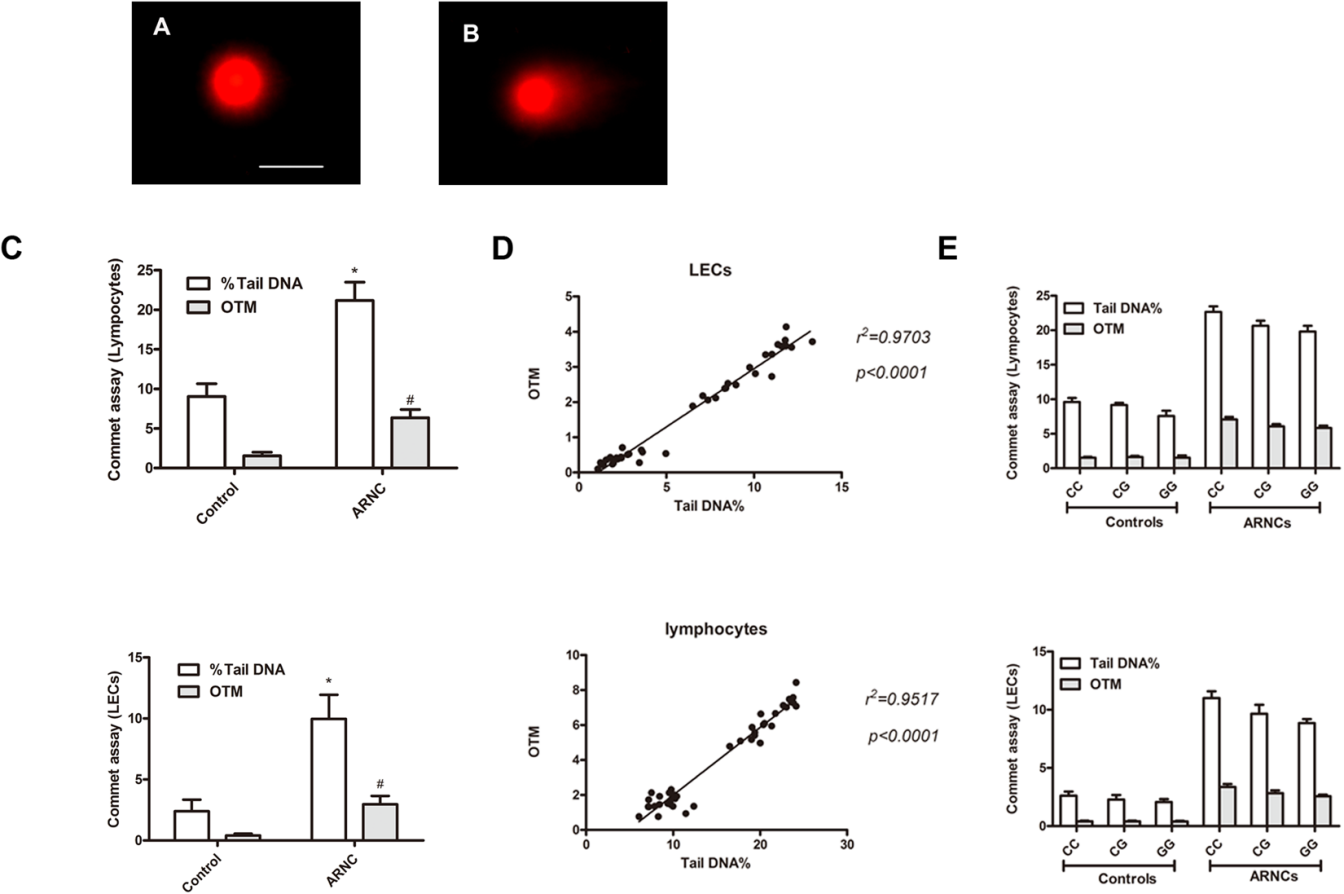
Representative images of comet assay: (**A**) controls and (**B**) ARNCs. (**C**) DNA breaks measured by Comet assay in LECs and peripheral lymphocytes, ARNCs had more DNA breaks than the controls. (**D**) The correlation between lymphocytes and LECs in %Tail DNA and OTM. (**E**) the DNA damage between different genotypes showed no difference. *P < 0. 001. Scale bars: 50 μm.

There were prominent comets indicating lots of DNA breaks in the LECs and peripheral lymphocytes of ARNCs and few in LECs and peripheral lymphocytes of the controls (Fig. 2A,B). The %Tail DNA and OTM by Comet assay in lymphocytes and LECs of ARNCs and controls are shown in Table 6. The assay showed there were much more DNA damage in ARNCs than controls (*P* < 0.001) (Fig. 2C). The %Tail DNA and OTM in LECs was positive correlation with those in lymphocytes (Fig. 2D). However, there no correlation of DNA breaks of peripheral lymphocytes and LECs with different genotypes of rs2229090 was found (Fig. 2E).

**Table 6:** DNA damage in LECs and lymphocytes of Controls and ARNCs.

Group	Lymphocytes		LECs	
	%Tail DNA	OTM	%Tail DNA	OTM
Controls	9.05 ± 1.60*	1.56 ± 0.44*	2.39 ± 0.95*	0.41 ± 0.15*
ARNCs	21.18 ± 2.30	6.37 ± 1.03	9.97 ± 1.97	2.97 ± 0.69

LECs: lens epithelial cells. *P < 0.001 in comparison with the Controls.

### Functional analysis of the rs2229090

**Figure 3:**
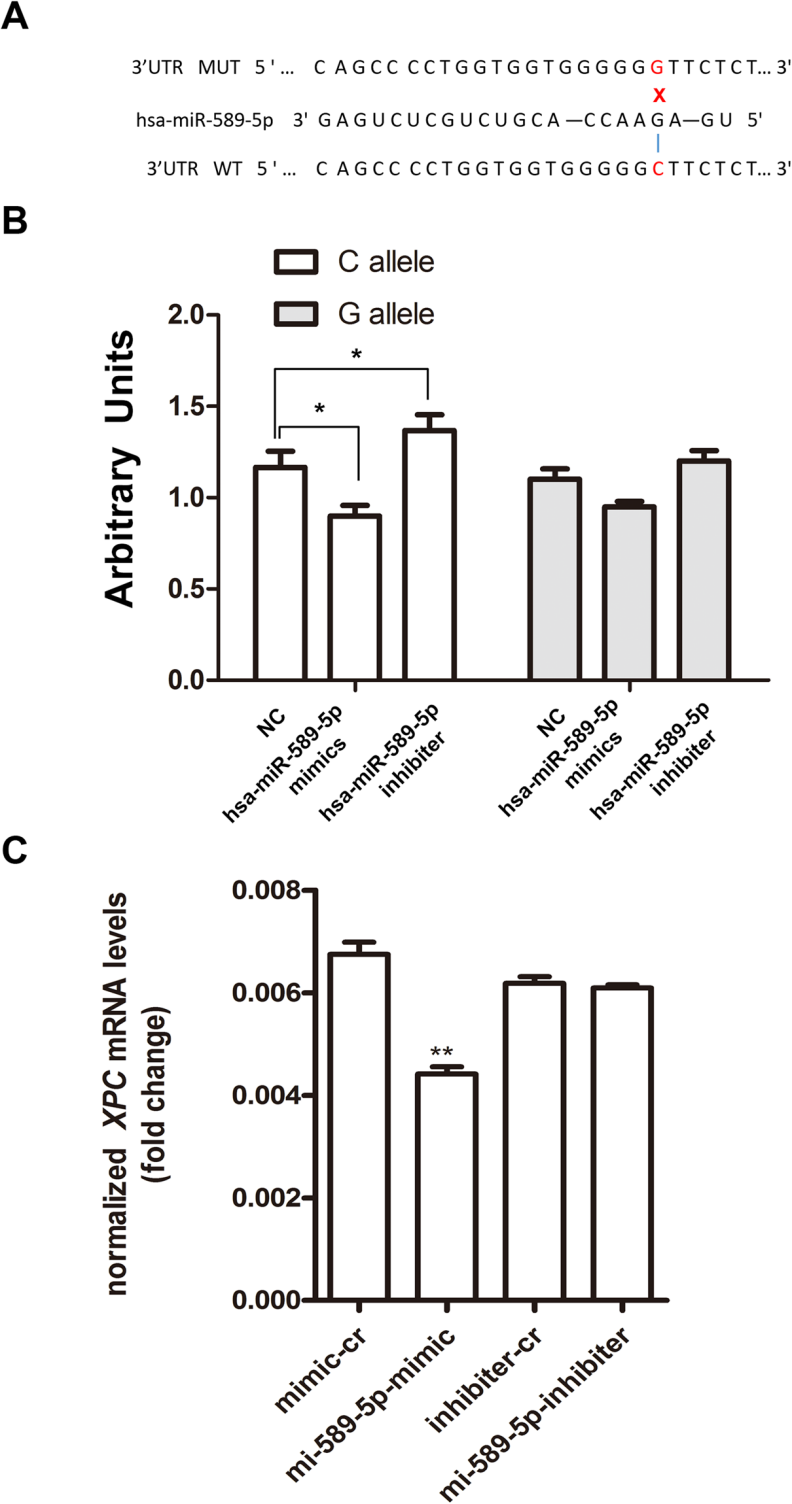
The effect of hsa-miR-589-5p on miRSNP rs2229090. (**A**) Constructs of different alleles of miRNA-binding sites. (**B**) MiR-589-5p mimics or inhibiters were co-transfected with the reporter constructs containing C allele or G allele into SRA01/04 cells. *P < 0.05, compared with G allele group. (**C**) Levels of *XPC* mRNA expression of the SRA01/04 cells transfected with hsa-miR-589-5p mimics or inhibitors. *P < 0.05, compared with control group.

The SNP rs2229090 is located in a putative 3′-UTR binding site of hsa-miR-589-5p, and The C allele of rs2229090 was predicted to bind more efficiently than the G allele to the miRNA (Fig. 3A).

To test whether there is an allele-specific effect of rs2229090 on *XPC* expression using a surrogate report gene in the presence of hsa-miR-589-5p, we transfected the miRNA mimics or inhibiters and constructed reporter plasmids (rs2229090-C and rs2229090-G) to SRA01/04 cell lines. Co-transfection with hsa-miR-589-5p mimics, the relative luciferase activity was lower in the reporter constructs containing rs2229090 C allelic than of the G allele in the cell lines. Meanwhile, co-transfection with hsa-miR-589-5p inhibitors, the relative luciferase activity was higher than the controls without hsa-miR-589-5p in the experiment using rs2229090 C allelic reporter constructs but not the G allelic reporter constructor (*P* > 0.05). (Fig. 3B), indicating that the interaction between hsa-miR-589-5p and the C allele of mRNA is more robust.

We further examined whether hsa-miR-589-5p could inhibit *XPC* expression in the cell line. We measured *XPC* mRNA after transfecting SRA01/04 cells (CC genotype) with has-miR-589-5p mimics and inhibitors., The *XPC* mRNA decreased when mimics were added as shown in Fig. 3C (P < 0.05).

## Discussion

DNA oxidative damage may lead to ARC, and its timely repair can maintain the healthy status in LECs^11^. DNA oxidative damage caused by various factors can be repaired through the DNA damage repair enzymes. Once the function of these repair genes is in malfunction, it would be a serious problem for cells and organisms^10^.

Many reasons can lead to the inefficiency of DNA repair, one of the reasons is the variation of DNA repair genes^34^. SNP are the most abundant form of DNA variation^13,14^. We have proved the roles of some vital genes of DSBR and NER in ARC^4,26^. In this study, we selected other genes of NER and DSBR pathways^10,19^ to explore whether they are also related to ARC formation. In the current research, we only found *XPC* -rs2229090 was associated with risk of ARNC with the C allele as a risk and G allele as a protection. This SNP is located in 3′-UTR region of the gene and in the binding sequence of hsa-miR-589-5p by in silico prediction. Hsa-miR-589-5p can reduce luciferase activity in an allele-specific manner (C allele) *in vitro* was found by luciferase reporter assays. We also found that the expression of *XPC* was lower in samples carry with the C allele in ARC and control groups. The mechanism of this genetic component to ARC pathogenesis could be described as: assuming that individuals have similar level of hsa-miR-589-5p in lens tissue; those with the C allele would have stronger interaction with hsa-miR-589-5p, resulting in lower XPC expression and DNA repair capability than the individuals carrying G allele.

NER is one of the most and well-established DNA repair mechanisms in maintaining genomic stability and integrity^35^. *XPC* is a vital part of the NER pathway and plays a vital role in the early steps of global genome NER^36,37^, which plays vital role for removal of oxidative damage^35^ and regulation of the cell cycle for DNA damage response^38^. The SNPs in the coding region of *XPC* have been associated with many diseases in some studies^39,40^.

MiRNAs participate in regulation of genes expression through the binding the 3′-UTR of target mRNA leading to mRNA degradation or translational repression^41^. Our data added new evidence on the biology of SNP and miRNA interaction in the context of gene expression Indeed, GG or CG genotype of rs2229090 was associated with significantly increased mRNA levels of *XPC* compared with CC genotype; the rs2229090 SNP has functional consequences on miRNAs targeting. We proved that the G allele of rs2229090 altered the expression of mRNA of *XPC*, which were highly possibly due to changes in binding free energy with hsa-miR-589-5p. In our previous study, compared with controls, ARC patients have more DNA damage in peripheral lymphocytes and in LECs. These damage of two locales were positively correlated^5^. In this study, the degree of DNA damage in peripheral lymphocytes and LECs assessed by Comet assay was significantly higher in ARNCs regardless of the genotypes. But the DNA damage between different genotypes showed no difference. Therefore, we believe that DNA damage in peripheral lymphocytes and LECs is common phenomenon and not associated with different allele in ARC group.

Currently, we could not explain why rs2229090 is exclusively associated with N types of ARC. Previous reports XPC binds to a wide variety of damage such as UV-induced photoproducts^42^. Our previous study showed that UV-induced DNA damage lead to the formation of N types of ARC^4^. In current study, we found the expression of XPC with C allele is lower in N types of ARCs than that of controls. We speculate that the lower expression of XPC leads to deficiency in repairing UV-induced damage, thus increasing the risk of ARNC.

In conclusion, our study focused on the importance of SNPs located in 3′-UTR of DNA repair genes to increase understanding of ARC pathogenesis. The results suggested that miRSNP rs2229090 of *XPC* may influence an individual’s susceptibility to ARNC in Han Chinese population. The rs2229090 C allele of *XPC* gene may be possive correlation with the risk of ARNC, and the mechanism may be that the free energy of hsa-miR-589-5p binging C allele is excessive than G allele of rs222900 of *XPC*, further destroy the post-transcription of *XPC*, thus leading to ARNC. This finding provides a novel approach and potential therapeutic target for ARNC management.

## Electronic supplementary material

Supplementary Dataset 1

## Data Availability

The datasets generated and/or analyzed during the current study are available from the corresponding author on reasonable request.
