# Supplementary material for: MicroRNA binding mediated Functional sequence variant in 3′-UTR of DNA repair Gene XPC in Age-related Cataract
Source: Sci Rep. 2018 Oct 12;8:15198. doi: 10.1038/s41598-018-33071-0 (PMC6185952; doi:10.1038/s41598-018-33071-0)
Supplement: Supplementary file 1 — Supplementary Dataset 1 [file 41598_2018_33071_MOESM1_ESM.docx]

**MicroRNA binding mediated Functional sequence variant in 3’-UTR of DNA repair Gene *XPC* in Age-related Cataract**

Xi Zou^1,2^, Lihua Kang^1^, Mei Yang^1^, Jian Wu^1^ and Huaijin Guan^1^

1, Department of Ophthalmology, Affiliated Hospital of Nantong University, Nantong, Jiangsu, China

2, The Third People's Hospital of Changzhou, Changzhou, Jiangsu, China

Corresponding author: Dr. Huaijin Guan, Department of Ophthalmology, Affiliated Hospital of Nantong University, 20 Xisi Road, Nantong, Jiangsu, China, Email: guanhjeye@163.com, Tel: +86-13809088972

**Table S1. The grade of lens opacity and identification codes of Controls and ARNCs**

| Controls | | | |  | ARNCs | | | |
| --- | --- | --- | --- | --- | --- | --- | --- | --- |
| Samples | Sex | Age(y) | LOCSⅢ |  | Samples | Sex | Age(y) | LOCSⅢ |
| No.1  No.2  No.3  No.4  No.5  No.6  No.7  No.8  No.9  No.10  No.11  No.12  No.13  No.14  No.15  No.16  No.17  No.18  No.19  No.20 | female  female  female  female  male  male  female  male  female  male  female  male  female  male  female  male  male  male  female  male | 65 | N0C1P0  N0C0P0  N0C0P1  N0C1P0  N0C0P0  N0C0P1  N0C1P0  N0C0P0  N0C1P0  N1C0P0  N0C0P1  N1C0P0  N1C1P0  N0C0P0  N0C0P1  N0C0P0  N0C0P0  N0C1P0  N0C0P0  N0C0P0 |  | No.1  No.2  No.3  No.4  No.5  No.6  No.7  No.8  No.9  No.10  No.11  No.12  No.13  No.14  No.15  No.16  No.17  No.18  No.19  No.20 | female  female  female  male  male  male  female  female  female  male  male  female  male  male  female  female  female  male  male  female | 58  58  75  75  55  72  62  72  61  65  72  60  65  64  71  76  72  55  65  62 | N3C0P0  N5C0P0  N4C0P0  N3C0P0  N5C0P0  N3C0P0  N3C0P0  N4C0P0  N3C0P0  N3C0P0  N5C0P0  N4C0P0  N3C0P0  N3C0P0  N3C0P0  N3C0P0  N5C0P0  N3C0P0  N4C0P0  N3C0P0 |
|  |  | 57 |  |  |  |  |  |  |
|  |  | 53 |  |  |  |  |  |  |
|  |  | 65 |  |  |  |  |  |  |
|  |  | 57 |  |  |  |  |  |  |
|  |  | 73 |  |  |  |  |  |  |
|  |  | 78 |  |  |  |  |  |  |
|  |  | 69 |  |  |  |  |  |  |
|  |  | 60 |  |  |  |  |  |  |
|  |  | 54 |  |  |  |  |  |  |
|  |  | 73 |  |  |  |  |  |  |
|  |  | 71 |  |  |  |  |  |  |
|  |  | 56 |  |  |  |  |  |  |
|  |  | 62 |  |  |  |  |  |  |
|  |  | 63 |  |  |  |  |  |  |
|  |  | 68 |  |  |  |  |  |  |
|  |  | 66 |  |  |  |  |  |  |
|  |  | 66 |  |  |  |  |  |  |
|  |  | 65 |  |  |  |  |  |  |
|  |  | 60 |  |  |  |  |  |  |
|  |  |  |  |  |  |  |  |  |
